# Ultrafast optical switching and data encoding on synthesized light fields

**DOI:** 10.1126/sciadv.adf1015

**Published:** 2023-02-22

**Authors:** Dandan Hui, Husain Alqattan, Simin Zhang, Vladimir Pervak, Enam Chowdhury, Mohammed Th. Hassan

**Affiliations:** ^1^ Department of Physics, University of Arizona, Tucson, AZ 85721, USA.; ^2^ Department of Material Science and Engineering, The Ohio State University, Columbus, OH 43210, USA.; ^3^ Ludwig-Maximilians-Universität München, Am Coulombwall 1, 85748 Garching, Germany.; ^4^ Department of Electrical and Computer Engineering, The Ohio State University, Columbus, OH 43210, USA.; ^5^ Department of Physics, The Ohio State University, Columbus, OH 43210, USA.

## Abstract

Modern electronics are founded on switching the electrical signal by radio frequency electromagnetic fields on the nanosecond time scale, limiting the information processing to the gigahertz speed. Recently, optical switches have been demonstrated using terahertz and ultrafast laser pulses to control the electrical signal and enhance the switching speed to the picosecond and a few hundred femtoseconds time scale. Here, we exploit the reflectivity modulation of the fused silica dielectric system in a strong light field to demonstrate the optical switching (ON/OFF) with attosecond time resolution. Moreover, we present the capability of controlling the optical switching signal with complex synthesized fields of ultrashort laser pulses for data binary encoding. This work paves the way for establishing optical switches and light-based electronics with petahertz speeds, several orders of magnitude faster than the current semiconductor-based electronics, opening a new realm in information technology, optical communications, and photonic processor technologies.

## Teaser

The attosecond optical switching and data encoding using ultrafast laser pulses is demonstrated.

## INTRODUCTION

**Fig. 1.**
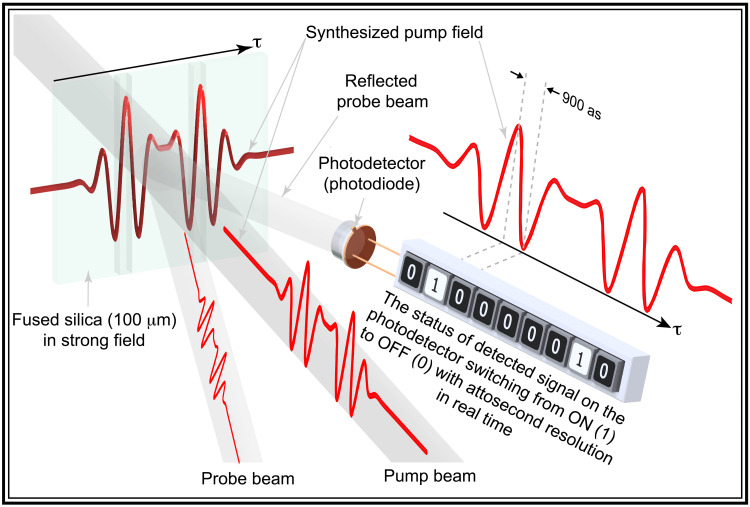
The basic principle of the attosecond optical switching based on the strong field interaction with dielectric. The pump light field induces the instantaneous reflectivity change in the dielectric (fused silica) system following the shape of the incident pump pulse waveform in real time. The reflectivity modification is detected by measuring the reflected probe beam’s change using a photodetector (e.g., photodiode) as a function of the time delay between pump and probe beams. The detected reflected signal is switched OFF/ON (presented by 0 and 1), depending on the field intensity at the time τ, in the real time. The switching resolution is equal to the duration of the half-cycle field (900 as) of the pump pulse and can be controlled by tailoring the pump field waveform using the attosecond light field synthesis approach. The attosecond optical switching and control allow to encode data on ultrafast laser pulse and open the door for establishing the ultrafast optical switches.

The strong light field interaction with the atomic system in the gas phase enabled the generation of the attosecond extreme ultraviolet (XUV) bursts by the means of high harmonic generation (HHG) (*1*–*3*). This advancement allowed the study and control of the electron motion induced by synthesized light fields (*4*, *5*). A revolutionary milestone was achieved by exploiting the strong field interaction to generate XUV pulses by HHG in bulk solids (*6*). The development of attosecond XUV (*7*–*11*) and attosecond all-optical spectroscopic measurements (*12*, *13*) provided an access to study and control the charge carrier dynamics and the optical properties of the condensed matter. For instance, the strong ultrafast laser pulses have been used to induce reflectivity and electronic structure modulation in dielectric material (*7*–*9*, *12*–*17*). In this case, the charge carriers are excited from the valance band to the conduction band in the dielectric system via multiphoton excitation (*17*). Then, the excited electrons in the conduction band move in the reciprocal space by acquiring a time-dependent momentum from the driving field (*7*, *12*, *13*, *17*, *18*). Hence, the electrons are accelerated and decelerated following the shape of the driver field’s vector potential, causing an instantaneous modulation in the electronic structure of the dielectric system (*12*, *18*–*20*). Consequently, the dielectric constant and the optical properties of the system are altered because of the strong polarizability (*15*, *21*). Accordingly, the field-induced reflectivity modulation of the dielectric systems following the driver field enables the control of the material and its optical properties in real time (*17*). Earlier, this interaction has been exploited for sampling the light field of laser pulses, demonstrating the capability of electron motion control, and determining the relative electronic delay response in different dielectric systems (*12*, *13*). In this work, we demonstrate the attosecond optical switching exploiting the oscillation of the dielectric material reflectivity from maximum to minimum in a half–field cycle time scale as illustrated in Fig. 1. Hence, the reflected light signal is switching from ON to OFF with subfemtosecond resolution. This demonstration paves the way to establish ultrafast optical switches with petahertz speeds beyond the state-of-the-art advancement of demonstrated optical switching (*22*–*34*). Moreover, we used complex synthesized light field waveforms of ultrafast laser pulses to control the switching signal and to demonstrate the digital binary ultrafast data encoding.

## RESULTS

### Transient reflectivity switching in subfemtosecond time scale

**Fig. 2.**
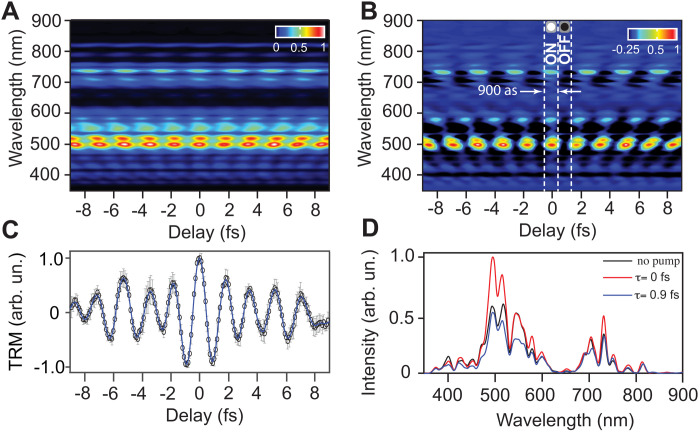
Attosecond optical switching. The reflectivity of SiO_2_ is modulated in real time due to the interaction with a strong (pump) light field. (**A**) The measured spectrogram (average of three scans) of the reflected probe beam as a function of the time delay between the pump and probe pulses. (**B**) The obtained spectrogram by subtracting the probe spectrum in the absence of pump field from the measured spectrogram [shown in (A)]. The reflectivity switches between maximum to minimum alternatively in 900-as time scale. (**C**) The normalized total reflectivity modulation (TRM) of the SiO_2_ in the strong field is retrieved from the measured spectrogram [in (A)] by the integration of the probe spectrum at each instance of time. (**D**) The probe beam’s spectrum reflected from the SiO_2_ in the equilibrium state (in the absence of pump field) is shown as the black line. In contrast, reflected spectra intensities of the probe beam [outlined from the spectrogram in (A)] at τ = 0 and 0.9 fs are plotted in the red and blue lines, respectively. arb. un., arbitrary units.

In our experiment, we use a synthesized light waveform generated by the attosecond light field synthesizer (ALFS) (*35*) (pulse duration of 2.7 fs) with a nominal carrier wavelength of 550 nm to modify the ultraviolet (UV)–grade fused silica (thickness of ~100 μm) reflectivity, which is probed by another weak light field (probe pulse), as explained in Materials and Methods. The reflected probe beam spectrum is recorded as a function of the time delay between the pump and probe pulses (Fig. 1). The measured spectrogram (average of three scans), depicted in Fig. 2A, shows the reflected probe beam (off the fused silica front surface) spectrum in real time. The reflectivity modulation is frequency and time dependent, which is distinctly observed (Fig. 2B) by subtracting the reflected probe spectrum in the absence of the driver field. As shown in the spectrogram in Fig. 2B, the reflectivity switches from maximum (ON) to a minimum (OFF) in subfemtosecond (900-as) time scale, showing an ultrafast optical switching speed of petahertz (1.1 PHz). The integration of the measured spectra amplitude as a function of time delay, total reflectivity modulation trace (Fig. 2C), gives an access to the vector potential and the pump field (see fig. S1) (*12*). At τ = 0 fs, the reflectivity of the fused silica increases by ~25% (the reflected spectrum of the probe beam, in this case, is shown as the red line in Fig. 2D) with respect to the reflectivity of the fused silica in the equilibrium state (the reflected spectrum with no field effect which is shown as the black line in Fig. 2D). In contrast, at τ = 0.9 fs, the reflectivity of the fused silica reduces by ~21% (blue line in Fig. 2D). Hence, the reflectivity changes by a total value of ~45% in a half-cycle time scale, demonstrating the switching intensity contrast. Moreover, the measured reflected spectra in Fig. 2D show that the strong field–induced reflectivity change of the fused silica is reversible following the driver field oscillation direction. Note that the transmission signal of the dielectric system is also varying in the strong field, although the transmitted light suffers nonlinear propagation and dispersion effects; therefore, the study of the transmitted light signal is complicated and does not solely reflect the induced dynamics of the system.

## DISCUSSION

**Fig. 3.**
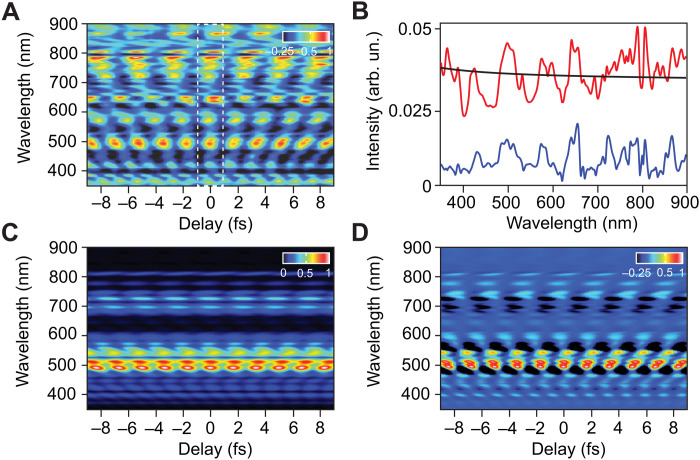
Simulated reflectivity dynamics of fused silica in a strong light field. (**A**) The retrieved reflectivity behavior of fused silica in strong field extracted from the measured spectrogram (see Discussion section) in frequency and time domains. (**B**) The amplitude of the reflectivity oscillation as a function of wavelength is shown as blue line calculated by halving the difference between maximum and minimum of an oscillation cycle [shown as white dashed rectangle in (A)]. The offset is calculated by averaging the maximum and minimum in an oscillation cycle and is shown as the red line. The transient reflectivity change of SiO_2_ under no influence of the pump pulse is shown as the black line. (**C** and **D**) The simulation of the measured spectrograms in Fig. 2 (A and B) are calculated by the developed simple model considering the effect of the spectral phase of the driver pulse as explained in the Discussion section.

The ultrafast reflectivity switching presented in Fig. 2 is due to the novel transient material modification controlled by intense synthesized light field in real time (*12*). The first step for proving and understanding the underlying physics of the frequency-dependent reflectivity switching in real time is to extract the modulated material reflectivity due to the strong light field interaction. This can be obtained by dividing the reflected probe pulse spectrum (black line in Fig. 2D) by the intrinsic reflectivity of fused silica and then deconvoluting the result from the measured spectrogram in Fig. 2A. The obtained spectrogram, plotted in Fig. 3A, represents the transient reflectivity change of the fused silica material induced by the strong field of the pump pulse in frequency and time domains.

From Fig. 3A, we extracted the average amplitude and offset of the reflectivity oscillation—in a full-cycle time frame, as highlighted with white dashed rectangle—at different frequencies and plotted in the blue and red lines in Fig. 3B, respectively. In addition, the reflectivity change of fused silica in the equilibrium state (no influence of the pump pulse) is plotted in the black line. The amplitude of the reflectivity oscillation at each frequency is calculated by halving the difference between the maximum (*I*_max_) and minimum (*I*_min_) intensity of a full cycle by [*I*_max_ − *I*_min_]/2. The offset at each frequency is calculated by averaging the maximum and minimum intensity in a full oscillation cycle by [*I*_max_ + *I*_min_]/2. Each amplitude value (blue line) determines the reflectivity modulation for that specific frequency, whereas the offset value (red line) shows the corresponding offset to the reflectivity value at the equilibrium state at a particular frequency.

This reflectivity modulations in Fig. 3A occur in the visible and flanking frequency ranges, which is similar to the spectral range of the pump pulse (from 370 to 900 nm) and far from its higher harmonics spectral range in the deep ultraviolet (DUV) and higher frequencies, indicating that the observed reflectivity modulation is not a result of the spectral interference between the fundamental and the HHG frequencies (*36*).

Furthermore, in the blue line in Fig. 3B, we can observe that the reflectivity modulation peaks oscillate over the entire spectral range from 370 to 900 nm, and these oscillations do not correlate with the maximum spectrum intensity peaks of the pump pulse spectrum (Fig. 2D, black line) and last longer than the pulse’s coherence time (*37*). Therefore, we can conclude that the observed reflectivity oscillation is not due to the optical interference between the pump and probe pulses (see section S2).

Moreover, it is clear in Fig. 3A that, for each frequency, the reflectivity is oscillating in a periodic oscillation in time associated with that frequency. In addition, the oscillation amplitude is controlled by the proximity to an individual resonance frequency in the excited dielectric permittivity induced by the intense pump field. The shearing features appear in the reflectivity spectrogram when the resonance frequencies are out of phase, which is the so-called “coherent dephasing.” The electric field of the pump pulse oscillates near zero value at the coherent dephasing time window, whereas the electric field of the pump pulse has substantial observable oscillations when the photon frequencies are coherently superimposed. Accordingly, the measured reflectivity modulation (shown in Figs. 2, A and B, and 3A) can be attributed solely to the transient change of the fused silica optical properties (i.e., susceptibility) in the strong field.

The susceptibility manifested by the collective behavior of dipole moments in dielectric can be described by the well-known Lorentz oscillator model (LOM) (*38*–*43*), which is widely applied for the understanding and analysis of the optical response for dielectric materials, including fused silica (*44*). The LOM assigns the Lorentz oscillators to major critical points in the joint density of states corresponding to the different interband transition energies, with extra oscillators modeling the absorption between these points (*44*). These critical points in unexcited fused silica exist at ≥10 eV, so there is no rapid reflectivity fluctuation induced by low intensity UV, visible (VIS), and near-infrared (NIR) light (see the black line in Fig. 3B). However, the blue line in Fig. 3B shows that the value of the reflectivity fluctuates rapidly in this wavelength range. Hence, to understand the underlying physics of this light induced reflectivity modulations, we developed a simple model that expresses the fused silica dielectric constant (ε), refractive index, and its reflectivity change in the strong field (for more details, see section S2).

The reflectivity modulation of the dielectric system in a strong field can be expressed as

$$R_{m}(\omega )=\frac{{\left[1-n(\omega )\right]}^{2}+\kappa^{2}(\omega )}{{\left[1+n(\omega )\right]}^{2}+\kappa^{2}(\omega )}$$ (1)

where $\tilde{n}=\pm \sqrt{{\tilde{\varepsilon }}_{r}}=n(\omega )+i\kappa (\omega )$ is the refractive index and ${\tilde{\varepsilon }}_{r}$ is the relative permittivity. For a particular frequency ω_0_, the electric fields of pump and probe pulses in the time domain can be expressed as

$$E_{\mathrm{pump}}=A_{0}e^{i\omega_{0}t}$$ (2)

$$E_{\mathrm{pro}}=A_{1}e^{i\omega_{0}(t+N\text{Δ}t)}$$ (3)

where *A*_0_ and *A*_1_ are the electric field amplitudes of the pump and probe pulses, respectively. In our experiment, *A* = *A*_0_/*A*_1_~10. Δ*t* is the time delay step (100 as), and *N*Δ*t* denotes the *N*th delay step.

After Fourier transformation, we can write Eqs. 2 and 3 in the spectral domain as

$${\tilde{E}}_{\mathrm{pump}}=A_{0}\delta (\omega -\omega_{0})$$ (4)

$${\tilde{E}}_{\mathrm{pro}}=A_{1}\delta (\omega -\omega_{0})e^{i\omega N\text{Δ}t}$$ (5)

and

$$\frac{{\tilde{E}}_{\mathrm{pump}}}{{\tilde{E}}_{\mathrm{pro}}}=Ae^{-i\omega N\text{Δ}t}$$ (6)

Assuming that the material polarizability, modified by the strong field interaction, affects the propagation of the probe pulse, so the electric field of the probe pulse can be expressed as

$$\nabla {\tilde{E}}_{\mathrm{pro}}=-\omega^{2}\mu_{0}(\varepsilon_{0}{\tilde{E}}_{\mathrm{pro}}+{\tilde{P}}_{\mathrm{pump}}+{\tilde{P}}_{\mathrm{pro}})=-\omega^{2}\mu_{0}\varepsilon_{0}{\tilde{E}}_{\mathrm{pro}}(1+{\chi_{1}}^{\prime }+{\chi_{2}}^{\prime })$$

$$=-\omega^{2}\mu_{0}\varepsilon_{0}{\tilde{\varepsilon }}_{r}{\tilde{E}}_{\mathrm{pro}}$$ (7)

where

$${\tilde{\varepsilon }}_{r}=1+{\chi_{1}}^{\prime }+{\chi_{2}}^{\prime }$$ (8)

$$\chi_{1}^{\prime }=Ae^{-i\omega N\text{Δ}t}\omega_{p}^{2}\sum_{k=1,2,\ldots }\frac{f_{k}}{{\omega_{0,k}}^{2}-\omega^{2}-i\omega {\text{Γ}}_{k}}$$ (9)

$$\chi_{2}^{\prime }=\omega_{p}^{2}\sum_{j=1,2,\ldots }\frac{f_{j}}{{\omega_{0,j}}^{2}-\omega^{2}-i\omega {\text{Γ}}_{j}}+C$$ (10)

and μ_0_ and ε_0_ are the permeability and permittivity in vacuum. ${\tilde{P}}_{\mathrm{pump}}$ is the pump-induced material polarization coupled to ${\tilde{P}}_{\mathrm{pro}}$, whereas the latter is the material polarization caused by the probe pulse. Equations 9 and 10 show that the susceptibility χ_1_′ (or χ_2_′) corresponding to ${\tilde{P}}_{\mathrm{pump}}$ (or ${\tilde{P}}_{\mathrm{prop}}$) can be expressed as a combination of multiple Lorentz resonators upon the pump excitation, where ω_0, *j*_, Γ*_j_*, and *f_j_* (or ω_0, *k*_, Γ*_k_*, and *f_k_*) are the natural frequency, damping rate, and strength of the *j*th (*k*th) resonator. *C* is a constant that represents the effect of resonances far from the spectrum range of interest. $\omega_{p}^{2}=\frac{e^{2}n_{e}}{m\varepsilon_{0}}$ is the square of the plasma frequency, and *m* is the free electron mass. Here, we assume one active electron per molecule in the fused silica: *n_e_* = 2.2 × 10^28^ m^−3^.

Accordingly, we simulate the experimentally measured reflectivity modulation spectrogram of fused silica (shown in Fig. 2A) using our pump pulse field (which is shown in fig. S1). The simulation results of the measured spectrograms (Fig. 2, A and B) can capture all the measured reflectivity modulation features, as shown in Fig. 3 (C and D, respectively), as explained in the Supplementary Materials. The obtained calculated spectrograms, shown in Fig. 3 (C and D), are in good agreement (SD of 1.37 and 2.1%, respectively) with the measured spectrogram in Fig. 2 (A and B). The fitting parameters for χ_2_^′^ and χ_1_^′^ are listed in tables S1 and S2.

On the basis of the experiment and theoretical results, the observed ultrafast reflectivity switching of fused silica can be attributed to the multiphoton resonances in the dielectric permittivity of the fused silica excited by our unique high-intensity and broadband near-single-cycle pump pulse. The novelty in our pump pulse is that it has strong field strength to induce the multiphoton excitation without damaging the fused silica system because it contains only 1.5 field cycles. Moreover, our pump pulse spans over 1.5 octaves, allowing for multiphoton excitation of fused silica with different photon combinations from the UV, VIS, and NIR spectral regions. In addition, the short pulse duration of the pump pulse (2.7 fs) implies that all the photons in the pump pulse are almost in phase, which is a key for inducing the reflectivity switching in the subfemtosecond time scale. Note that the weak intensity optical pulse will not induce a multiphoton excitation; thus, no temporal reflectivity modulation would be observed.

In addition, the presented reflectivity modulation spectrograms in Fig. 2 (A and B) carry the signature of the broadband pump pulse spectral phase dispersion. Therefore, the presented experiment can be used as an accurate methodology for characterizing the ultrashort laser pulses and its spectral phase dispersion directly and with a high resolution (see fig. S2B), which is beyond the capability of the typical ultrashort pulse characterization techniques (e.g., frequency-resolved optical gating). Moreover, this transient modification can be controlled to engineer the pump pulse waveform (spectral phase) to achieve a tunable refractive index of natural material (i.e., fused silica), which, before this work, was only possible in metamaterials (*45*, *46*), opening the door for a vast range of applications in ultrafast photonics.

### Ultrafast optical information encoding

**Fig. 4.**
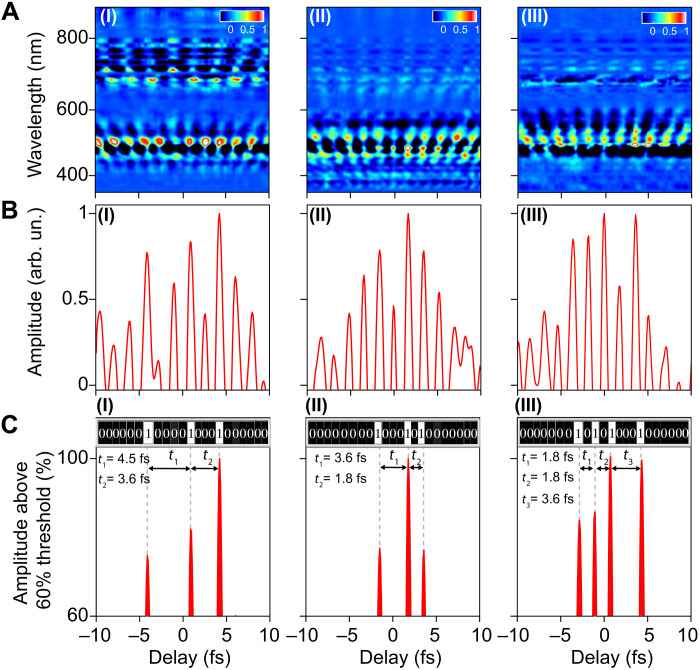
Ultrafast light field encoding. (**A**) (I to III) The measured spectrograms of the reflected probe beam triggered by three different synthesized waveforms after subtracting the probe spectrum in the absence of the pump field. (**B**) (I to III) The positive value of the probe spectra integration as a function of time, representing the measured light signal by a photodetector in real-time, after subtracting the background. The light signal switches ON/OFF alternatively every half-cycle. (**C**) (I to III) The detected light signals above a 60% threshold. The light signals are switched ON and OFF at different time intervals. In the insets, the slots present the signal’s detection status in real time as follows: Black (0) means that no signal was detected above the threshold, while white (1) means the signal is above the threshold and seen by the detector. This control of the optical switching signal would enable the binary data encoding on light fields with petahertz speed.

As demonstrated experimentally in Fig. 2, the light-induced phase transition of the fused silica allows us to switch between an ON and OFF state of the reflected light signal following the driver field. Consequently, the reflectivity modulation and the switching alterability can be controlled by tailoring the driver field waveform. Next, we demonstrate the control of the switching signals using on-demand synthesized complex waveforms generated by ALFS (*5*, *35*, *47*). Figure 4A (I to III) shows some of the measured reflectivity modulation spectrograms, after subtracting background spectrum, triggered by three different synthesized light fields. The corresponding integrated intensities of the reflected spectra at different instances of time (above zero amplitude) are plotted in Fig. 4B (I to III). Note that the light signal can also be measured by photodiode detector instead of the spectrometer to directly detect the integrated intensity signal. The light signal switches from ON to OFF states uniformly every half-cycle of the driver field. By setting a certain amplitude threshold (60%) in Fig. 4B (I to III), which easily can be experimentally implemented or programmed in a photodetector, the number of the detected light signals (above this threshold) and the switching alternative time vary depending on the shape of the driver waveform. Figure 4C (I to III) shows the signals above the 60% threshold, and the insets in the top (contains 22 slots) represent the signal status (OFF or ON) in black and white in real time at each half-cycle of the driver field. Using the first waveform, the signal switches ON and OFF three times with a time separation of 4.5 and 3.6 fs Fig. 4CI. This switching time interval is controlled to be 3.6 and 1.8 fs (as shown in Fig. 4CII) using the second waveform. Moreover, the number of the switching signal increases to four signals by using the third waveform with 1.8, 1.8, and 3.6 fs time period separations between the signals as shown in Fig. 4CIII.

This capability of controlling the light signal switching (ON/OFF) allows the ultrafast data encoding with synthesized light waveforms (*35*), which are beyond the reach by conventional ultrafast pulses field. Accordingly, the reflected signal above the threshold will be detected “ON status” and presents the binary code “1.” The reflected signal below the threshold—will not be detected by the photodetector and hence will have an “OFF status”—represents the binary code “0.” The number of coding bits that the light field can carry equals twice the number of the driver light field cycles. Some of the examples of binary encoding using the synthesized waveforms are shown in the insets of Fig. 4C (I to III).

In a potential ultrafast light field encoding process (illustrated in fig. S4), the data will be encoded on the synthesized light waveforms generated by the ALFS (*35*) (or any pulse shaping device), which will act as an “encoder” device. Then, the synthesized waveform (which is the encoded laser beam) will carry the data from the transmitter to the receiver station. Next, the encoded laser beam will be focused on the dielectric together with another beam (decoder laser beam). Last, the reflected decoder laser beam from the dielectric will be detected by a photodetector. After setting a certain predefined threshold, the photodetector will read the coded data in the 1 and 0 binary form. For simplifying the encoding process, a simple narrow band probe (decoding) pulse can be used because switching speed and data encoding resolution depend only on the half-cycle duration of the pump pulse.

Notably, the number of encoded binaries depends on the number of the field cycles within the pump pulse, while the encoding bitrate is defined by the pump laser repetition rate. Therefore, a multicycle pulse will carry more binary information. Although, a high-power multicycle pulse will be required for triggering the reflectivity switching. In this case, the damage threshold of the fused silica will be less, and the number of the cycles (binaries) in the encoding pulse is limited.

Furthermore, this demonstrated that optical switching occurs under ambient conditions, allowing a simple realistic architecture of a potentially realistic compact optical switch integrated on a photonic chip (*48*). Moreover, the data encoding on ultrafast light waveforms, in contrast to the encoding provided by modern electronic sources using a microwave, would notably enhance the data processing and transformation speed for light-time distances.

In conclusion, the light field–induced phase transition of dielectric system in strong field enables switching the reflected light signal ON and OFF with attosecond switching speed. The light field tailoring and shaping with high resolution allow us to demonstrate the ultrafast optical switching control and data binary encoding by synthesized laser pulses. This work paves the way to develop ultrafast optical-based switches and to transfer data with petahertz speed and beyond, which can carry information to the deep space and open a new era in communication and information technology.

## MATERIALS AND METHODS

### Time-resolved reflected spectra measurements

A multicycle pulse, carried at a central wavelength of 800 nm, is focused and propagated in a hollow-core fiber to generate a broadband spectrum that spans from UV to NIR spectral regions. This supercontinuum is divided into three spectral channels and compressed inside the ALFS apparatus (*35*). At the exit of ALFS, the three channels are superimposed to generate a synthesized waveform of 2.7-fs pulse. The relative delays and intensities of the three channels inside the ALFS are controlled to synthesize complex waveforms on-demand. The carrier-envelope phase of the synthesized waveform is passively locked to less than 100 mrad [the laser source is optical parametric chirped-pulse amplification (OPCPA) based]. In addition, the relative phases between the three channels’ pulses inside the ALFS are actively locked to ensure the waveform’s stability during the experiment (*35*). The output beam from the ALFS is divided into two beams by passing through a two-hole mask with different hole diameters. One of the two merged beams has a high intensity (estimated field strength is 1 V/Å) and is used as a pump beam to alter the reflectivity of the dielectric system (*12*). The second beam (probe beam) has a lower intensity (~2.5% of the pump beam intensity) so it is not inducing any reflectivity changes on the system. Notably, the pump and probe pulses have the same waveforms (merged from one input pulse). The two beams are focused and overlapped on the UV-grade fused silica sample (thickness of ~100 μm) by two focusing D-shape mirrors (*f* = 10 cm). One of the two D-shape mirrors is attached to a high-resolution (nanometer) delay stage. The probe beam is partially reflected from the front face of the fused silica sample. Then, it is filtered out from the pump beam (at the far field) and focused into the entrance of an optical spectrometer, so the reflected beam from the second surface is not contributing to the measured spectra. The reflected probe beam’s spectra are acquired as a function of the time delay between the pump and probe pulses. Moreover, the complex waveforms used to control the reflectivity and control the switching signals (shown in Fig. 4) are generated by changing the relative phase delay and intensities between the ALFS channels [more details about the light field synthesis scheme by ALFS can be found elsewhere; (*35*)]. Moreover, the sampling of the arbitrary waveforms used in the demonstration of the light field encoding experiments is performed with the same setup and at the same light field sampling position. Notably, the potential photodiode for the ultrafast encoding applications shall have a high speed and broad flat bandwidth response, which covers from the DUV to NIR frequencies.

## Supplementary Materials

### This PDF file includes:

Sections S1 to S3 Figs. S1 to S4 Tables S1 and S2
