# Supplementary material for: Ultrafast optical switching and data encoding on synthesized light fields
Source: Sci Adv. 2023 Feb 22;9(8):eadf1015. doi: 10.1126/sciadv.adf1015 (PMC9946343; doi:10.1126/sciadv.adf1015)
Supplement: Supplementary file 1 — Sections S1 to S3 Figs. S1 to S4 Tables S1 and S2 [file sciadv.adf1015_sm.pdf]

Science Advances
AAAS

Supplementary Materials for

Ultrafast optical switching and data encoding on synthesized light fields

Dandan Hui *et al.*

Corresponding author: Mohammed Th. Hassan, mohammedhassan@arizona.edu

Sci. Adv. **9**, eadf1015 (2023)
DOI: 10.1126/sciadv.adf1015

This PDF file includes:

Sections S1 to S3
Figs. S1 to S4
Tables S1 and S2

1- The pump pulse utilized to induce the attosecond optical switching in dielectric fused silica

We utilized the measured spectrogram in Fig. 2A to retrieve the electric field of the pump pulse from the TRM depreciated in Fig. 2A as we previously demonstrated (9).

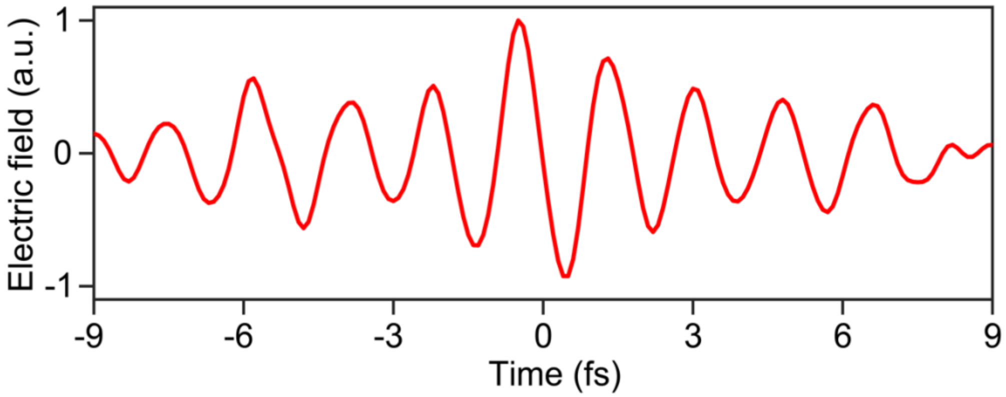

Fig. S1. Electric field driving attosecond optical switching in fused silica. The retrieved driver electric field from the TRM trace (shown in Fig. 2A in main text) is used to induces the phase transition and the reflectivity modulation shown in Fig. 2A in main text. This field has been also used in the simulation presented in Fig. 3, C and D.

2- The simulation model and the calculations of the fused silica reflectivity modulation in strong field

As we discussed in the article, the interference between the pump and probe pulses cannot contribute to the reflectivity modulation shown in the measured spectrograms in Figs. 2 and 3. The frequency-dependent period suggests another form of interference may be the key. Since the reflectivity of a material is directly determined by its susceptibility, material polarization driven by the strong field can be a possible source of this interference effect. The susceptibility manifested by the collective behavior of dipole moments in dielectric can be described by the well-known Lorentz Oscillator Model (LOM) (38-43), which is widely applied for the understanding and analysis of the optical response for dielectric materials, including fused silica (44). The LOM assigns the Lorentz oscillators to major critical points in the joint density of states corresponding to the different interband transition energies, with extra oscillators modeling the absorption between these points (44). The LOM also suggests that the frequency-dependent reflectivity peaks are associated with Lorentz oscillators corresponding to the critical points. The resonance frequency is at the climbing edge of the reflectivity peak; the height and width of the climbing edge are determined by the strength and damping rate of the oscillator. These critical points in unexcited fused silica exist at and beyond 10 eV, and therefore, if low intensity 10-12 eV light is incident on fused silica, strong reflectivity variation will be in that wavelength range due to the presence of the critical points (49). For low intensity

pump pulse, there is NO rapid reflectivity fluctuation (indicated by the black curve in Fig. 3B). Moreover, the duration of the induced polarization field is similar to that of the pump pulse. Since the pump pulse is much shorter than the coherence time, the polarization field cannot interfere with the probe pulse. Hence, the interference between the polarization field and probe pulse has no contribution to the reflectivity oscillation as a function of time.

However, Fig. 3B shows that, in our case, the value of the reflectivity is not slowly varying in the spectro-temporal domain at all. Instead, it oscillates in real time with the amplitude and offset fluctuating rapidly as a function of the wavelength. Therefore, the measured reflectivity oscillation is attributed to strong-pump-induced critical points in fused silica, which causes these strong (over ±20%) ultrafast (900 as) reflectivity modulations in fused silica. Typically, the number of oscillators applied in the LOM ranges from four to six (44). In our calculation, we apply seven oscillators covering the spectral range in the measurement. For wavelengths from short to long, the resonance frequencies correspond to photon energies 3.49, 3.02, 2.50, 2.18, 1.94, 1.64, and 1.38 eV, respectively, approximately equivalent to 3, 4, 4, 5, 5, 6, and 7 incident photons at the center wavelength of the pump pulse required for multi-photon transitions between the valence and conduction bands. These wavelengths are obtained by calculations based on the physical model that will be described in detail later. Hence, the source of these emerging major critical points is very likely the N-photon absorption (N = 3, 4, 5, 6, 7) induced by the intense pump pulse. As a result, the frequency-dependent amplitude and offset of the reflectivity modulation are controlled by the collective impact of the N-photon absorption channels. The N-photon absorption should be transient because *ex-situ* measurements found no observable change in the irradiated fused silica. Nevertheless, the relaxation of N-photon excitation driven by the strong field should take much longer than the photon re-emission (reflected probe pulse) process without our proposed transition process, especially in the case of weak pump irradiance. In other words, when below the damage threshold, the irradiated fused silica is able to retain a short memory of the part of the intense few-cycle pulse that participates in the N-photon absorption and eventually recovers itself. Assuming the relaxation time is much longer than the coherence time, in this case, the polarization field caused by the N-photon absorption can effectively interfere with the probe pulse and the probe-induced polarization field, causing the steady and observable reflectivity oscillation within the delay range shown in Figs. 2 and 3.

Figure 3A shows another intriguing phenomenon, the horizontally shearing features in the reflectivity spectrogram, which can be explained by the so-called coherent dephasing in the pump pulse. The coherent dephasing originates from the spectrally dependent phase of the ultrashort pulse; that is to say, the phase does not remain constant in the pulse spectrum but varies with frequency. Based on the above discussion, assuming that the reflectivity oscillation is caused by the interference among the pump-driven polarization field, the probe pulse, and the probe-induced polarization field, the spectrally dependent phase of the pump and probe pulses will also be reflected in the interference, adding the same amount of phase shift to the oscillation at the corresponding frequency. Suppose the phase of the pulse fluctuates spectrally.

In that case, the phase shift of reflectivity oscillation will fluctuate accordingly, which appears as the asymmetric shearing feature in Fig. 3A. This relationship is intuitively presented in fig. S2 in the Supplement.

The reflectivity modulation spectrogram shown in Fig. 2A can be modeled by utilizing Eqs. 9 and 10. In order to verify the proposed relationship between the coherent dephasing of the pump pulse and the shearing features in the reflectivity spectrogram, we extracted the frequency-dependent phase delay in the reflectivity spectrogram (fig. S2(A)) and applied it as the spectral phase profile of the pump pulse.

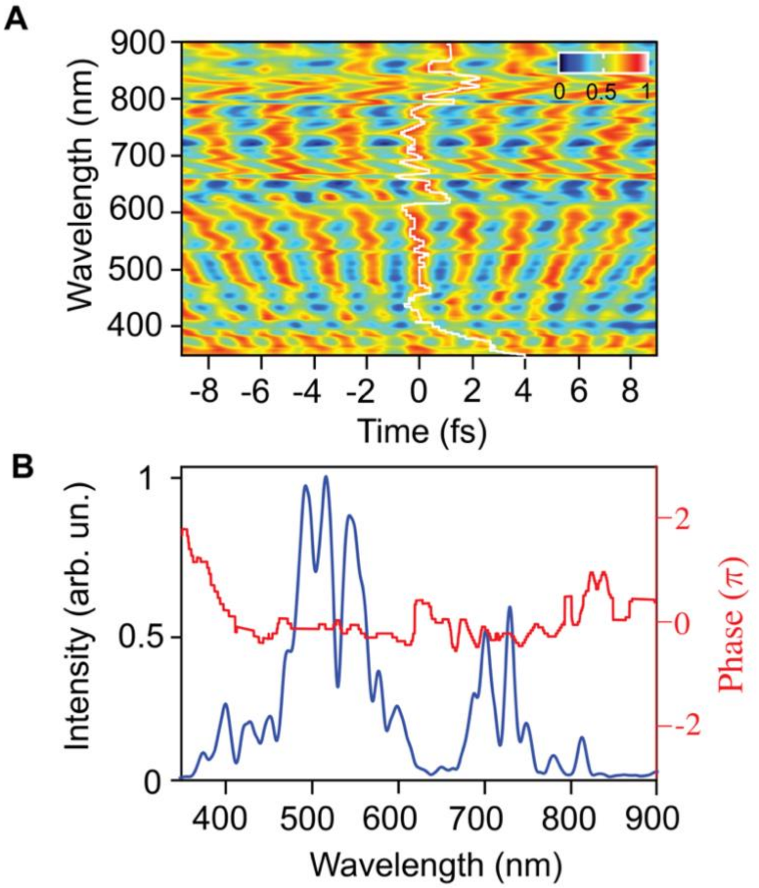

Fig. S2. The effect of the driver pump pulse phase dispersion on the reflectivity oscillation. (A) Normalized measured spectrogram shown in Fig. 3 in the main text. The white line trace shows the spectral phase effect on the reflectivity modulation. **(B)** The measured spectra (solid blue curve) and the retrieved spectral phase from A is plotted in redline.

In our calculations, we used a temporal profile of the pump pulse (fig. S1) obtained from the experiment as the input for modeling. Seven resonators are used in Eqs. 9 and 10 (in the article) for each to model the multiple-resonance effect and temporal oscillation in Fig. 3A. The modeling parameters are listed in tables S1 and S2 for χ_2' and χ_1', respectively.

Table S1. Parameter for calculation of $\chi_2{}'$ (C=0.9)

Resonance	1	2	3	4	5	6	7
Photon energy (eV)	3.49	3.02	2.50	2.18	1.94	1.64	1.38
Photon number	3	4	4	5	5	6	7
MPI energy (eV)	10.47	12.08	10.00	10.90	9.70	9.84	9.66
f_j	0.0227	0.0102	0.0079	0.0027	0.0023	0.0034	0.0018
Γ_i ($\times 10^{14}$ s^{-1})	4.0	4.0	2.5	2.0	2.0	2.0	2.0

Table S2. Parameter for calculation of $\chi_1{}'$

Resonance	1	2	3	4	5	6	7
Photon energy (eV)	3.49	2.88	2.50	2.18	1.94	1.64	1.38
Photon number	3	4	4	5	5	6	7
MPI energy (eV)	10.47	11.52	10.00	10.90	9.70	9.84	9.66
f_k ($\times 10^{-4}$)	5.48	3.99	3.99	2.49	1.99	1.99	1.20
Γ_k ($\times 10^{14}$ s^{-1})	4.0	4.0	2.5	2.0	2.0	2.0	2.0

Figure 3A suggests that the asymmetry in reflectivity oscillation across the spectrum very likely originates from the phase drift of each frequency component of the pump pulse. This is because Eq. 9 is the only term in Eq. 8 that can introduce an oscillation to the reflectivity, and Eq. 9 is associated with the phase of each monochromatic wave that comprises the ultrashort pump pulse. The calculated spectrograms shown in Fig. 3, C and D are consistent with the measurements shown in Fig. 2, A and B. The major characteristics in the measured reflectivity modulation, including the shearing feature, resonances in the visible wavelength range, and the oscillation versus the delay, are all captured in the simulation result. These small damping rates also support our hypothesis, explaining the high contrast of the interference between $\tilde{P}_{pump}$ and $\tilde{P}_{pro}$ within the 20-fs delay window. Moreover, in fig. S3, we plotted the calculated frequency-dependent reflectivity in contrast with the measured results shown in Fig. 3B. The excellent agreement between the calculation and measurement strongly supports our proposed hypothesis.

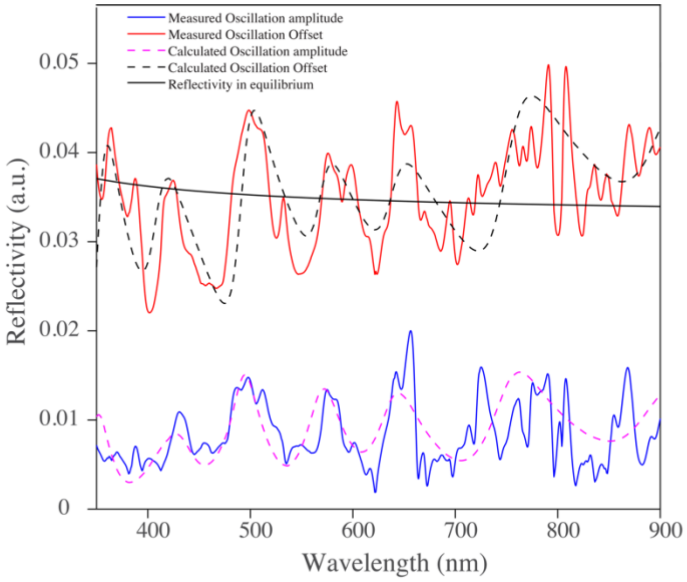

Fig. S3. Comparison of the frequency-dependent reflectivity between modeling and measurement. The measured reflectivity and retrieved oscillation amplitude and offset are shown in blue and red lines. The corresponding calculation are shown in dotted black and violet lines.

3- Ultrafast optical information encoding application

The potential scheme for ultrafast data encoding is shown in fig. S4. The input data in the binary form (0 & 1) will be sent to the light field synthesizer or the pulse shaper (encoder unit) device to tailor the field accordingly and encode the input data on the light field. The laser beam travels from the coder to the decoder unit. The data will be decoded based on measuring the reflectivity modulation of the dielectric.

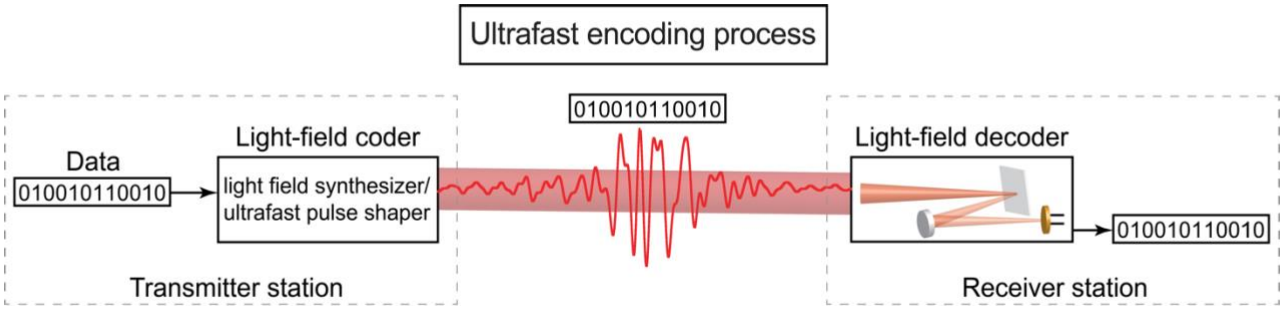

Fig. S4. Ultrafast data encoding. Schematic of potential ultrafast light field encoding process exploiting the demonstrated optical switch with synthesized waveforms.
